# Face-ism and Objectification in Mainstream and LGBT Magazines

**DOI:** 10.1371/journal.pone.0153592

**Published:** 2016-04-13

**Authors:** Nathan N. Cheek

**Affiliations:** Department of Psychology, Swarthmore College, Swarthmore, PA, United States of America; Macquarie University, AUSTRALIA

## Abstract

In visual media, men are often shown with more facial prominence than women, a manifestation of sexism that has been labeled *face-ism*. The present research extended the study of facial prominence and gender representation in media to include magazines aimed at lesbian, gay, bisexual, and transgender (LGBT) audiences for the first time, and also examined whether overall gender differences in facial prominence can still be found in mainstream magazines. Face-ism emerged in *Newsweek*, but not in *Time*, *The Advocate*, or *Out*. Although there were no overall differences in facial prominence between mainstream and LGBT magazines, there were differences in the facial prominence of men and women among the four magazines included in the present study. These results suggest that face-ism is still a problem, but that it may be restricted to certain magazines. Furthermore, future research may benefit from considering individual magazine titles rather than broader categories of magazines, given that the present study found few similarities between different magazines in the same media category—indeed, *Out* and *Time* were more similar to each other than they were to the other magazine in their respective categories.

## Introduction

The representation of gender in the media has interested psychologists and other researchers for many decades (for a review, see [1]). Studies often find that women are represented less positively than men across different types of media (e.g., [2–6]). For example, women are often featured less prominently and portrayed as more submissive, and there is often more attention paid to the physical appearance of women [2, 6]. Research on media representation is important because such representation can be internalized, which can then lead to self-objectification, body dissatisfaction, and eating disorders, among other consequences [1, 7–8]. Moreover, media representations can perpetuate stereotypes, thereby potentially continuing the cultural derogation of negatively perceived groups [1].

Because many researchers have realized the importance of investigating the content and form of gender representation in the media, several different approaches have been developed to study empirically how men and women are portrayed. For instance, analyses can focus on specific behaviors of people in television shows, advertisements, and magazines (e.g., how many times characters of different genders eat [4]), or they can be more visually-oriented, focusing instead on poses, clothing, or other visual elements of representation (e.g., [9]). In the present research, I adopted one such visual approach—namely, the comparison of the facial prominence of men and women in two “mainstream” magazines (i.e., magazines that are aimed at a general audience and tend to reflect dominant societal views; see, e.g., [10]) and in two magazines aimed at lesbian, gay, bisexual, and transgender (LGBT) audiences.

### Facial Prominence and Face-ism

More than 30 years ago, Archer, Iritani, Kimes, and Barrios [11] explored what they called *face-ism*: the tendency for men to have more facial prominence—measured by dividing the length of people’s faces by the combined length of their faces and bodies in an image—than women in photographs, paintings, drawings, and other visual representations. They found that women were lower in facial prominence than men in magazines from the U.S. and eleven other countries, as well as in artwork spanning 600 years. Subsequent studies have also found evidence of face-ism in television commercials [12], television shows [13], and even in pictures of politicians on the Internet [14–16]. In each of these studies, men had more facial prominence than women.

Gender differences in facial prominence have important implications because they may perpetuate stereotypes and influence person perception. Archer and colleagues [11] claimed that gender differences in the prominence of different parts of the body reflect differences in the value placed on different attributes of men and women. They argued that because Western societies traditionally value men’s intellect, more prominence is given to men’s faces, whereas the relative prominence of women’s bodies communicates the value placed on their physical appearance instead of their intellect. Moreover, Frederickson and Roberts [7] note that body prominence is inherently objectifying because it suggests that women can be represented through their physical appearance: “the visual media portray women as though their bodies were capable of representing them” (p177). Experimental studies have also shown that observers attribute less mental activity and morality to people with less facial prominence; in other words, they are literally perceived as more object-like than people with more facial prominence [17].

Face-ism represents more than a visual manifestation of sexism, however, because facial prominence also influences impression formation, such that images with more facial prominence create more positive impressions. In their initial research, Archer and colleagues [11] created two sets of photographs of the same subjects with different degrees of facial prominence. They found that participants rated the same people as more intelligent and ambitious when they were pictured with more facial prominence. Other studies have also found that people are perceived as more intelligent, competent, and dominant when featured with high facial prominence [18–19], and Schwarz and Kurz [20] suggested that higher facial prominence has the more general effect of increasing positive impressions and likeability. Because higher facial prominence leads to better impressions, face-ism in the media may not only reflect stereotypical views of men and women, but actually reinforce such views as well, in addition to generally presenting men in a more positive light. Hence, understanding how facial prominence varies across images in magazines is important not only because it may reveal how different people are portrayed in the media, but also because it may shed light on the impressions drawn from media representations.

### Face-ism in the 21^st^ Century

Although many recent studies have documented face-ism in online pictures (e.g., [14–16]), it may be that overall gender differences in facial prominence have disappeared in magazine photos as society has grown more gender equal over time. Indeed, Matthews [21] coded photographs from several mainstream magazines published in 2004 and found that, overall, men and women did not differ in facial prominence. Matthews notes, however, that these findings should not be interpreted as suggesting that sexism no longer shapes the representations of men and women in the media; rather, it may just manifest itself more subtly, at least with regard to facial prominence. Specifically, Matthews found that when occupation was taken into account, gender differences did emerge, though not always as predicted by original research. When images in mainstream magazines showed people in intellectual careers (e.g., politicians, doctors, etc.), men indeed had more facial prominence than women. In contrast, when images showed people in more physical careers (e.g., construction workers, athletes, etc.), this gender difference reversed: men actually had *less* facial prominence than women. Matthews concluded that the specific attributes that are valuable to a particular career are highlighted more in men—when intellect is important, men have more facial prominence than women, whereas when more physical attributes are important, men have more body prominence than women.

Matthew’s [21] results appear to suggest that face-ism, if not completely gone from mainstream magazines, is at least less easily observable than before. However, when Melkote and Melkote [22] examined photographs from issues of *Newsweek* published in 2005, they found face-ism as expected: men had more facial prominence than women in photographs. Moreover, their comparison between *Newsweek* photographs from 2005 and 1985 did not reveal any decline in face-ism, suggesting that face-ism has not even decreased, much less disappeared. Thus, these two studies present conflicting results, and one goal of the present research was to investigate whether overall gender differences in facial prominence can still be observed in mainstream magazines.

### The Present Research

The present study had three goals. First, given the contradictory results of the studies by Matthews [21] and Melkote and Melkote [22], I sought to further investigate face-ism in mainstream magazines to determine if overall gender differences in facial prominence would still emerge. The present research does not address the role of occupation in predicting facial prominence, because, in order to find photographs of physical careers, Matthews did not follow Archer and colleague’s [11] guidelines for image selection (see method section for details) and included photographs designed to capture particular movements or body parts (e.g., a soccer player kicking a ball). In contrast, the present study applied all of Archer et al.’s guidelines, and thus it was unclear how any conclusions about occupation from the present study would relate to those of Matthews, given that different rules were used for image selection. The present sample of photographs may include more depictions of people in intellectual careers than in physical careers due to the magazines from which the photographs originated (e.g., *Newsweek* as opposed to *Sports Illustrated*). It is worth noting, however, that other studies have found that women have less facial prominence even in physical occupations [23], and thus future search should continue to explore the potential influence of occupation on facial prominence.

Second, no previous research has examined face-ism in LGBT magazines, but substantial research has shown that the portrayal of both men and women in LGBT magazines differs considerably from gender representations in mainstream magazines [24–27]. Accordingly, it may be that patterns of gendered facial prominence will be different in LGBT magazines. For example, common stereotypes about homosexuality often assume that lesbians are more like heterosexual men and gay men are more like heterosexual women [28], and to the extent that gender differences in facial prominence in mainstream magazines reflect stereotypical views of heterosexual men and women (e.g., [11]), it may be that gendered patterns of facial prominence in LGBT magazines reflect stereotypical views of gay men and lesbians. Thus, the pattern of gender differences in facial prominence may actually be reversed in LGBT magazines. Of course, not all men and women depicted in LGBT magazines are homosexual, but it may be that this stereotype is pervasive enough to influence gender representation in general.

Finally, the third goal of this study was to examine differences in objectification between mainstream and LGBT magazines. Some previous comparisons of mainstream and LGBT magazines have suggested that there is less objectification in LGBT magazines (see, e.g., [25]), though others have concluded the opposite (see, e.g., [24, 26]), Because degree of facial prominence can be conceptualized as a measure of objectification [7, 11, 17], a comparison of the facial prominence of men and women in mainstream and LGBT magazines may help shed light on debates about patterns of objectification in magazines aimed at different audiences.

## Method

### Image Selection

This study included 918 photographs featured in articles and advertisements from two mainstream (*Time* and *Newsweek*) and two LGBT (*The Advocate* and *Out*) magazines. Two hundred thirty-seven photographs were coded from 14 issues of *Time*, 226 photographs were coded from 15 issues of *Newsweek*, 263 photographs were coded from 15 issues of *The Advocate*, and 192 photographs were coded from 19 issues of *Out*. *Time* and *Newsweek* were chosen both because they are aimed at a general audience and focus generally on news and culture and because they have been studied extensively in previous face-ism research (e.g., [11, 21–22]). *The Advocate* and *Out* were chosen because they are aimed at a general LGBT audience (as opposed to a specific group such as transgender people) and also focus on news and culture; hence, their broad goals roughly match those of *Time* and *Newsweek*, although they are intended for a different audience.

Photographs were selected in accordance with the following criteria established by Archer and colleagues [11]:

(a) photographs may contain only one human subject, (b) photographs that aim to capture some particular body region, movement, problem, or gesture cannot be counted (e.g., performing athletes, people modeling clothes or cosmetics, using tools, in weight loss ads, etc.), (c) photographs with a “co-subject” cannot be used (a person next to an elephant, a driver with a car, etc.), and (d) photographs printed several times are counted only once (photographs of regular columnists, entertainment ads, etc.), and (e) photographs of disembodied heads are not counted (p727).

These guidelines are intended to limit the extent to which photographs are biased towards certain parts of the body or other factors that would impede a clear analysis of general facial prominence. For the purposes of the present study, coders were told to code any transgender people they encountered as the gender they presented, and photographs of transgender people were not coded or analyzed separately.

Two primary coders, blind to the purpose of the present study, randomly selected 918 photographs from the four magazines of interest. After image selection was complete, two blind reliability coders checked the photographs selected by the primary coders and confirmed that the guidelines were followed.

### Face-ism Index

This study followed the initial analytic strategy designed by Archer and colleagues [11] and used in the vast majority of facial prominence research. For each photograph, a *face-ism index* was computed by first measuring the length of just the face in the image, then measuring the length of the face and body in the image, and finally dividing the former by the latter. Face-ism indices thus represent the prominence of the face relative to the body in a photograph (rather than the face relative to the total area of the photograph) and can theoretically range from 0 (an image featuring only someone’s body) to 1 (an image featuring only someone’s face).

### Reliability of Face-ism Index Measurement

To ensure reliability of coding, one of the two primary coders computed the face-ism index for each of the photographs, and one of the two reliability coders later coded a subset of photographs to determine measurement reliability. One reliability coder coded a subset of 142 photographs coded by the first primary coder. In line with previous studies, there was a high correlation between the two coders’ judgments, *r* = .98, *p* < .001, suggesting strong reliability of measurement. The other reliability coder coded a subset of 46 photographs coded by the second primary coder. Once again, there was a high correlation between the two coders’ judgments, *r* = .97, *p* < .001.

## Results

Descriptive statistics are presented in Table 1. To investigate gendered patterns of facial prominence and possible differences in objectification between mainstream and LGBT magazines, I conduced a 2 (magazine type: mainstream vs. LGBT) X 2 (gender: men vs. women) ANOVA. There was no main effect of magazine type *F*(1, 914) = .02, *p* = .899, *η*_*p*_^*2*^ = .00, nor was there a significant interaction between gender and magazine type, *F*(1, 914) = .94, *p* = .333, *η*_*p*_^*2*^ = .00. There was a marginally significant main effect of gender, *F*(1, 914) = 3.17, *p* = .076, *η*_*p*_^*2*^ = .00. I then conducted a 4 (magazine title: *Time* vs. *Newsweek* vs. *The Advocate* vs. *Out*) X 2 (gender: men vs. women) ANOVA to more closely examine patterns of facial prominence across all four magazines. There was a small but significant main effect of gender, *F*(1, 910) = 4.03, *p* = .045, *η*_*p*_^*2*^ = .00, a significant main effect of magazine title, *F*(1, 910) = 23.51, *p* < .001, *η*_*p*_^*2*^ = .07, and a significant interaction, *F*(1, 910) = 3.54, *p* = .014, *η*_*p*_^*2*^ = .01.

**Table 1:** Face-ism Indices of Men and Women in Mainstream and LGBT Magazines.

	Men			Women		
Magazine	*n*	Face-ism Index	Standard Deviation	*n*	Face-ism Index	Standard Deviation
**Mainstream Magazines Combined**	254	.48	.23	209	.44	.21
***Time***	140	.39	.20	97	.40	.21
***Newsweek***	114	.58	.22	112	.47	.21
**LGBT Magazines Combined**	227	.46	.22	228	.45	.21
***The Advocate***	141	.50	.22	122	.50	.20
***Out***	86	.41	.21	106	.39	.21

**Fig 1:**
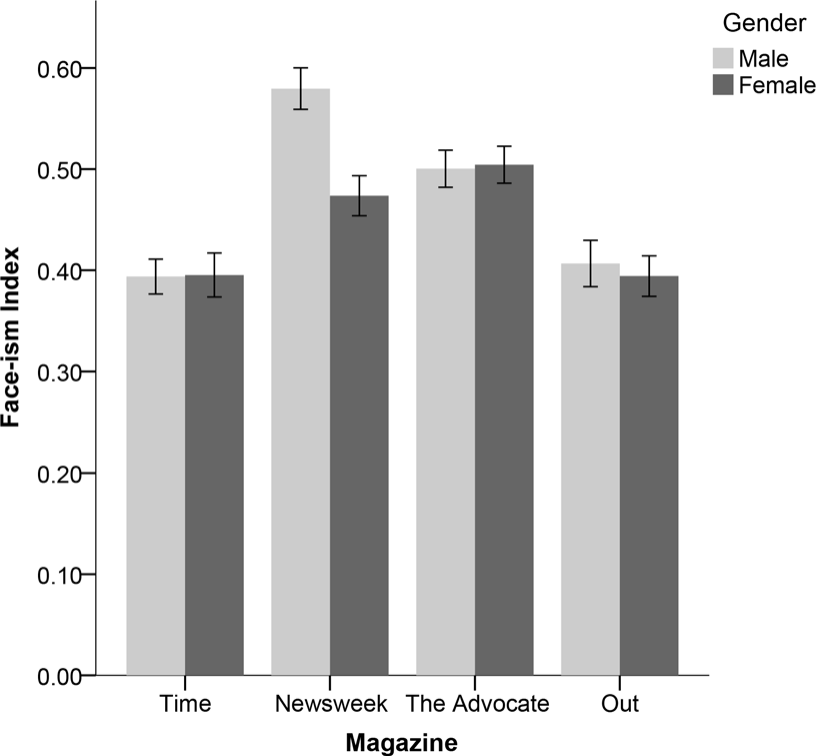
Face-ism Indices of Men and Women in Two Mainstream and Two LGBT Magazines. Error bars represent the standard error of the mean.

Because examination of the means presented in Table 1 suggested that magazines within the same category (e.g., *Time* and *Newsweek*) did not always exhibit the same patterns of facial prominence, thus complicating the interpretation of the interaction between magazine title and gender, I conducted exploratory post hoc Bonferroni contrasts comparing all eight cells of the present study design (i.e., men and women in each of the four magazines). Contrasts revealed that there were no differences in facial prominence among men and women in *Time* and men and women in *Out* (all *p*’s > .999). Men and women in *The Advocate* and women in *Newsweek*, none of whom differed from each other in facial prominence (all *p*’s > .999), had higher facial prominence than both men and women in both *Time* and *Out* (all *p*’s < .034). Finally, men in *Newsweek* had higher facial prominence that men and women in *Time* and *Out* (all *p*’s < .001), as well as women in *Newsweek* (*p* = .005). Men in *Newsweek* did not differ in facial prominence from men (*p* = .085) and women (*p* = .175) in *The Advocate*. Fig 1 illustrates how the facial prominence of men and women varied across the four different magazines.

## Discussion

In the present study, I compared the facial prominence of men and women in mainstream and LGBT magazines to investigate (1) whether mainstream magazines still exhibit face-ism, (2) whether face-ism exists in contemporary LGBT magazines, and (3) whether studying facial prominence could shed light on questions about the relative level of objectification in mainstream and LGBT magazines. In contrast to the findings of Matthews [21], overall gender differences in facial prominence did emerge in *Newsweek*, suggesting that although face-ism may be less prevalent than before (e.g., there was no evidence of face-ism in *Time*), women’s bodies still receive more focus than men’s in at least some mainstream magazines [22]. There was no evidence of face-ism in either *Out* or *The Advocate*, and thus it may be that LGBT magazines in general have more equal representations of men and women; however, more magazine titles would need to be examined to test this possibility more thoroughly.

Somewhat surprisingly, magazines within the same category (i.e., mainstream or LGBT) exhibited few similarities with regard to facial prominence. Indeed, the facial prominence of men and women in *Out* and *Time* did not differ, whereas there was greater facial prominence in *The Advocate* and *Newsweek* than in *Out* and *Time*, respectively. This pattern of results calls into question conclusions about differences between broad categories or genres of magazines made in previous research [24–27], because it may be that, at least with facial prominence, there is little within-category coherence among difference magazine titles. Thus, future research may benefit from focusing on specific magazine titles in addition to comparing broadly-defined genres or categories of media.

It is worth noting that although degree of facial prominence can serve as one operationalization of objectification [7, 11], there are other ways to analyze the portrayal of bodies in media. For example, Saucier and Caron [26] found that LGBT magazines tend to contain almost exclusively images of muscular men (see also [24]), and argued that these magazines promote unrealistic images of men and also place emphasis on the physical appearance of men over their other attributes, a pattern that could also fall under the category of objectifying media representations. Thus, future research might continue to investigate how images in both LGBT and mainstream magazines objectify men and women using other analytic approaches, though future studies should examine individual magazine titles in addition to broad media categories.

### Implications

A more complete understanding of patterns of facial prominence in the media is important because research has shown that people internalize the values and images to which they are exposed; for instance, people who are frequently exposed to images of people with low levels of facial prominence may be more likely to post pictures of themselves that also have a relatively large emphasis on their bodies relative to their faces. Reichart Smith and Cooley [29] surveyed social network profile pictures in seven different countries and found consistent evidence of what could be called “self-inflicted face-ism”: women generally had less facial prominence in their self-chosen profile pictures than men. Although there may be many factors contributing to this pattern, one important influence appears to be magazine exposure: Kapidzic and Martins [30] found that more magazine exposure predicted greater internalization of media values (e.g., the value placed on women’s bodies and men’s intellect), which in turn predicted lower levels of facial prominence in participants’ Facebook profile pictures. Thus, exposure to (in this case, mainstream) magazines may play a role in how social media users present themselves online.

Although there might, in theory, be no reason why pictures with more body prominence are worse than pictures with more facial prominence, it is worth noting that, as mentioned in the introduction, several studies have shown that when person perceivers engage in impression formation, they tend to judge images of people with less facial prominence less positively [11, 18–20]. Accordingly, it is possible that exposure to face-ism in the media and subsequent internalization of gendered patterns of facial prominence may undermine people’s—particularly women’s—impression management by leading to less positive perceptions from observers. Of course, future research is needed to more closely examine this possibility, but past research on the role of facial prominence in person perception does highlight why face-ism can be potentially harmful.

An implication of the present study is that readers of *Out* and *Time* may be exposed to lower levels of facial prominence in general, such that they may internalize the value placed on bodies and physical appearance by the media more than readers of *The Advocate* and *Newsweek*. Moreover, readers of *Newsweek* may be more likely to internalize and perpetuate gender stereotypes as a result of differences in the facial prominence of men and women. Future investigations could explore these possibilities by examining the influence of exposure to specific magazines on stereotype endorsement and self-objectification.

Future research on face-ism should also consider the causes of face-ism in addition to why images with more facial prominence create more positive impressions [11, 18–20]. For instance, one possible cause of face-ism is that objects of the *male gaze* [31–32], whereby women or other groups are represented and visually coded with reference to male audiences, tend to be presented with lower facial prominence. It may be that face-ism is more pervasive in mainstream magazines because the male gaze is more directly focused on women, whereas in LGBT magazines, the male gaze is more diffuse, shaping the visual portrayal of both men and women. Similarly, it may be that facial prominence is perceived positively because it is associated with other typically masculine attributes (e.g., agency, intellect) that society privileges over more stereotypically feminine attributes (e.g., passivity, beauty) [11]. This possibility would suggest that researchers should also investigate potential interventions to change this pattern of person perception, because although it might be beneficial in the short run for certain groups to strategically harness the impression boost of higher facial prominence, in the long run, it may be more beneficial to move toward reducing the influence of relative face or body prominence in general.

### Limitations

Although the results of the present research may have interesting and potentially important implications, there are notable limitations. First, only two titles of each type of magazine were included in analyses; future research could include different magazines to increase the generalizability of the present findings. Second, this study focused only on gender, without regard to race, age, or other demographic variables. Given that previous research has found that people of different races [5, 33–34] and ages [35] receive different media portrayals (often in line with common stereotypes), it will be important for future research to include such variables in analyses of facial prominence. Indeed, race, age, gender, and other attributes like sexual orientation all intersect, and thus understanding not only how individual factors can influence facial prominence but also how multiple factors can interact to shape media representation is essential.

An additional limitation of the present study is that the only genders considered were male and female, despite the fact that there are many other ways people identify (e.g., trans*, genderqueer). This issue is particularly relevant given that LGBT magazines are more likely to represent people with different gender identities than mainstream media. Because marginalized populations tend to have less facial prominence in general (perhaps because of lower social status) [33], it seems possible that although cisgender men and women have equal degrees of facial prominence in LGBT magazines, transgender and non-binary queer people may have relatively less facial prominence. Thus, future research could examine more closely how gender identity influences facial prominence in media images.

Finally, future research could also look back at previous issues of LGBT magazines from the past few decades to investigate possible past gender differences in facial prominence. Previous research [21]—as well as the present study—suggests that face-ism used to exist widely in mainstream magazines and has diminished (though not completely disappeared), but a limitation of the present research is that it is unknown whether face-ism also used to exist in LGBT magazines, or if there has always been gender equality in facial prominence in LGBT magazines. Hence, archival studies on possible changes (or lack thereof) in facial prominence of men and women in LGBT magazines could help shed light on this open question.

## Conclusion

In conclusion, the present study examined the facial prominence of men and women in mainstream and LGBT magazines, and the results suggest that overall gender differences in facial prominence are still present in at least some mainstream media. That only one gender difference emerged across the four magazines suggests that there may be less overt sexism in the media now than there was 30 years ago, though it is worth noting that face-ism has not disappeared in other contexts, such as online profiles of politicians [14–16] or users of social networking sites [29]. In addition, the results suggest that future research should focus more on individual magazines in addition to broader categories or genres. Overall, the present study highlights the potential usefulness and implications of using facial prominence as an analytic approach to the study of visual images, and helps underline the importance of continued attention to face-ism, sexism, and other forms of inequality in the media.

## Supporting Information

S1 File Face-ism Data. This contains the face-ism data for the 918 coded photographs.
(SAV)

S2 File Reliability Data Part 1. This contains the reliability data for the first primary coder’s data.
(SAV)

S3 File Reliability Data Part 2. This contains the reliability data for the second primary coder’s data.
(SAV)
